# Antiretroviral therapy and Kaposi’s sarcoma trends and outcomes among adults with HIV in Latin America

**DOI:** 10.1002/jia2.25658

**Published:** 2021-01-06

**Authors:** Jessica L Castilho, Ahra Kim, Cathy A Jenkins, Beatriz Grinsztejn, Eduardo Gotuzzo, Valeria Fink, Denis Padgett, Pablo F Belaunzaran‐Zamudio, Brenda Crabtree‐Ramírez, Maria Mercedes Escuder, Rosa Alencar Souza, Simone B Tenore, Sidnei R Pimentel, Maria Letícia Rodrigues Ikeda, Paulo R de Alencastro, Unai Tupinanbas, Carlos Brites, Estela Luz, Juliana Netto, Claudia P Cortes, Alexandre Grangeiro, Bryan E Shepherd, Catherine C McGowan, the Caribbean, Central, South America network for HIV Epidemiology (CCASAnet)

**Affiliations:** ^1^ Division of Infectious Diseases Vanderbilt University Medical Center Nashville TN USA; ^2^ Department of Biostatistics Vanderbilt University Medical Center Nashville TN USA; ^3^ Instituto Nacional de Infectiologia Evandro Chagas Fiocruz Brazil; ^4^ Universidad Peruana Cayetano Heredia Instituto de Medicina Tropical Alexander von Humboldt Lima Peru; ^5^ Fundación Huésped Investigaciones Clínicas Buenos Aires Argentina; ^6^ Instituto Hondureño de Seguridad Social and Hospital Escuela Universitario Tegucigalpa Honduras; ^7^ Deparatmento de Infectologia Instituto Nacional de Ciencias Médicas y Nutrición Salvador Zubirán. Mexico City Mexico; ^8^ São Paulo State Department of Health Institute of Health São Paulo Brazil; ^9^ São Paulo State Department of Health AIDS Reference and Training Center São Paulo Brazil; ^10^ Care and Treatment Clinic of the Partenon Sanatorium Rio Grande do Sul State Department of Health Porto Alegre Brazil; ^11^ University of Vale do Rio dos Sinos São Leopoldo Brazil; ^12^ Medical School Federal University of Minas Gerais Belo Horizonte Brazil; ^13^ Edgar Santos University Hospital Complex Federal University of Bahia Salvador Brazil; ^14^ Fundaciòn Arriaran and University of Chile School of Medicine Santiago Chile; ^15^ Department of Preventive Medicine University of São Paulo School of Medicine São Paulo Brazil

**Keywords:** Kaposi’s sarcoma, HIV, antiretroviral therapy, incidence, mortality, Latin America

## Abstract

**Introduction:**

Kaposi’s sarcoma (KS) remains the most frequent malignancy in persons living with HIV (PWH) in Latin America. We examined KS trends and outcomes from Latin American clinical sites in the era of increased access to antiretroviral therapy (ART).

**Methods:**

Cohorts in Brazil, Peru, Mexico, Honduras, Argentina and Chile contributed clinical data of PWH ≥16 years old from 2000 to 2017, excluding patients with KS diagnosed before clinic enrolment. We compared KS incidence over time using multivariable incidence rate ratios. Predictors of KS before/at or after ART initiation and of mortality after KS were examined using Cox regression.

**Results:**

Of 25 981 PWH, 481 had incident KS, including 200 ART‐naïve and 281 ART‐treated patients. From 2000 to 2017, the incidence of KS decreased from 55.1 to 3.0 per 1000 person‐years. In models adjusting for CD4 and other factors, the relative risk for KS decreased from 2000 to 2008. Since 2010, the adjusted risk of KS increased in the periods before and ≤90 days after ART initiation but decreased >90 days after ART. In addition to low CD4 and male‐to‐male sex, KS risk after ART was associated with age and history of other AIDS‐defining illnesses. Mortality after KS (approximately 25% after five years) was not associated with either year of KS diagnosis nor timing of diagnosis relative to ART initiation.

**Conclusions:**

KS incidence in Latin America has remained stable in recent years and risk is highest before and shortly after ART initiation. Early diagnosis of HIV and ART initiation remain critical priorities in the region.

## 1 INTRODUCTION

Kaposi’s sarcoma (KS) is a frequent type of cancer among persons living with HIV (PWH) in Latin America [1]. In the United States, KS incidence dramatically fell with the introduction of combination antiretroviral therapy (ART) [2]. However, the risk of KS has remained elevated in Latin America and its epidemiology in the current ART era has not been thoroughly described [1, 3]. In Latin America, ART was introduced and made first available to PWH with the lowest CD4 cell counts in 1996 in Brazil, Mexico, Argentina and Chile and in 2000 to 2004 in Honduras and Peru. Access was gradually expanded over time to include those with CD4 cell counts <350 cells/μL in 2004 to 2010, <500 cells/μL in 2008 to 2014, and lastly to all PWH regardless of CD4 cell count in 2014 to 2015.

Globally, KS has been strongly associated with severe immunodeficiency, male sex (particularly men who have sex with men [MSM]), and regional prevalence of human herpes virus 8 (HHV‐8) [4, 5]. Among patients on ART, HIV viral load and cumulative measures of viraemia over time have also been associated with increased risk of KS, suggesting HIV viral replication itself plays a role in KS pathogenesis [6]. While survival following KS diagnosis has improved with ART, PWH with a history of KS have an increased risk of mortality [7, 8, 9]. Patient characteristics associated with mortality after KS have not been well described.

This study examines the epidemiology, risk factors and outcomes of incident KS in the current ART era among PWH in Latin America using a multinational cohort.

## 2 METHODS

### 2.1 Study design, settings and data management

This study utilized data from two multi‐cohort, observational studies of adults living with HIV in Latin America: the Caribbean, Central and South America network for HIV Epidemiology (CCASAnet) and Coorte Brasil. CCASAnet includes clinical sites from seven Latin American countries and is a member of the International Epidemiologic Databases to Evaluate AIDS (IeDEA) consortium [10]. CCASAnet sites with cancer data participated in this study: Instituto Nacional de Infectologia Evandro Chagas (Rio de Janeiro, Brazil); Fundación Arriarán (Santiago, Chile); Instituto Hondureño de Seguridad Social and Hospital Escuela Universitario (Tegucigalpa, Honduras); Instituto de Medicina Tropical Alexander von Humboldt, Universidad Peruana Cayetano Heredia (Lima, Peru), Centro Medico Huesped (Buenos Aires, Argentina) and Instituto Nacional de Ciencias Médicas y Nutrición, Salvador Zubirán (Mexico City, Mexico). Coorte Brasil is a multi‐site observational cohort of adults initiating ART in Brazil during 2003 to 2014 [11]. Seven Coorte Brasil sites collaborated with CCASAnet to validate clinical endpoints including KS and were included in this analysis: Instituto Nacional de Infectologia Evando Chagas (Rio de Janeiro) (included in the analysis within CCASAnet); AIDS Reference and Training Center (São Paulo); São Paulo State Municipal Health Department – Santana (São Paulo); São Paulo State Municipal Health Department – São Jose do Rio Preto (São Jose do Rio Preto); Care and Treatment Clinic of the Partenon Sanatorium (Porto Alegre); Federal University of Minas Gerais (Belo Horizonte); and Edgar Santos University Hospital Complex (Salvador). Demographic, clinical and laboratory data were collected at every site, de‐identified and sent to the CCASAnet Data Coordinating Center at Vanderbilt University (Nashville, USA) for data harmonization and processing. The data were checked for internal consistency and missing data, and quality assessments were performed. Institutional ethics review boards from all sites and Vanderbilt approved the project, waiving the requirement for individual patient informed consent.

PWH ≥ 16 years of age enrolled in a CCASAnet or Coorte Brasil site between January 2000 and December 2017 were included in this study. KS can occur in children and adults with HIV. This study focused on adults and used age criteria consistent with other global studies of cancer epidemiology in PWH [4]. We excluded patients with KS diagnoses occurring prior to cohort entry. Only the first KS diagnosis after cohort entry was included in analyses. Validated KS diagnoses were based upon clinical exam findings and/or histological and radiographic findings as documented in the medical records. Patients contributed person‐time until date of death, last clinic visit if a gap in care of >12 months occurred, or date of database closure for patients in ongoing clinical care.

### 2.2 Temporal trends in KS incidence

We calculated KS incidence per calendar year and adjusted incidence rate ratios using multivariable general estimating equations including calendar year, clinical site, age at cohort entry, sex/sexual route of HIV infection (heterosexual men, heterosexual women, MSM, other/unknown men and other/unknown women), CD4 cell count at cohort entry (square root transformed) and prevalent AIDS‐defining illness (excluding KS) at cohort entry. Calendar year, age and CD4 cell count at enrolment were fit with restricted cubic splines (four knots) to relax assumptions of linearity [12]. To examine KS incidence relative to ART initiation, we repeated the analysis stratifying for person‐time before ART initiation (from cohort entry to initiation of ART or censoring/death for those who never started ART), within the first 90 days of ART initiation, and after the first 90 days of ART. KS diagnosed on the day of ART initiation was considered to have occurred before ART initiation. Missing data (CD4 cell count and sex/sexual risk) were imputed using multivariable imputation with chained equations with 10 imputation replications. Imputation models included all covariates in the main model as well as the interaction of the outcome (Kaposi’s sarcoma) and the person‐years of follow‐up.

### 2.3 Risk factors associated with KS relative to ART initiation

We used Cox proportional hazard models to examine patient characteristics associated with KS before and after ART initiation among ART‐naïve patients. In these analyses, observation time was again divided into before ART initiation and after ART initiation. In the first analysis, age, history of other AIDS‐defining illness, sex/sexual risk factor for HIV transmission, HIV RNA (logarithmic transformed) and CD4 cell count (square root transformed) at clinic entry were evaluated in univariate and multivariable Cox models for KS risk from date of clinic entry to date of ART initiation. Observation time was censored at first occurrence of date of KS diagnosis, ART initiation, death or loss to follow‐up. In the second analysis, the above patient characteristics and ART regimen class associated with KS risk following ART initiation were evaluated from the date of ART initiation to KS diagnosis, death or loss to follow‐up. All Cox models were stratified by country. Age, HIV RNA and CD4 cell count were fit using restricted cubic splines with three knots. Missing data (CD4 cell count, HIV RNA, sex/sexual risk) were multiply imputed using chained equations with ten imputations. Imputation models included all covariates in the main model as well as the interaction of the outcome (Kaposi’s sarcoma) and the person‐time of follow‐up.

### 2.4 Mortality risk following KS

Lastly, we evaluated whether the timing of KS diagnosis relative to ART initiation was associated with mortality. We included all patients with incident KS. Patients with ART treatment before clinic enrolment were considered to have KS after ART. Patients who never started ART were considered to have KS before ART. We examined whether patient characteristics at KS diagnosis (age, sex/sexual risk factor for HIV transmission, CD4 cell count, HIV RNA and calendar year) and timing of KS diagnosis (before or after ART initiation) were associated with mortality risk using Kaplan–Meier curves and multivariable Cox proportional hazard models stratified by country. Age and CD4 cell count (square root transformed) were fit using restricted cubic splines with three knots. Missing data (CD4 cell count, HIV RNA, sex/sexual risk) were multiply imputed using chained equations with 10 imputation replications. Imputation models included all covariates in the main model as well as the interaction of the outcome (death) and the person‐time of follow‐up.

All analyses were completed using R version 3.5.3 (2019‐03‐11). Statistical code for all analyses is available at http://biostat.mc.vanderbilt.edu/ArchivedAnalyses.

## 3 RESULTS

### 3.1 Cohort characteristics

In total, 27 425 PWH were included in CCASAnet and Coorte Brasil cohorts between 2000 and 2017. Of those, 257 (0.9%) were excluded for KS diagnoses before clinic enrolment. Our analysis included a total of 25 981 PWH who contributed 133 233 person‐years of follow‐up. Of these, 78% were ART‐naive at clinic entry. There were 481 diagnoses of KS at or after clinic entry, including 107 (22%) diagnosed on the date of entry, and 145 (30%) diagnosed within 30 days following entry. Two hundred patients were diagnosed with KS before ART initiation, 125 were diagnosed within the first 90 days after ART initiation, and 156 were diagnosed more than 90 days following ART initiation. Among those who developed KS before ART, the median time from clinic entry to KS was five days (interquartile range [IQR]: 0 to 28 days). Among those who developed KS after ART, the median time from ART initiation to KS was 92 days (IQR: 28 days to 1.09 years).

As shown in Table 1, PWH with incident KS were more likely to be from Brazil, Mexico and Chile and to be MSM. They had lower median CD4 cell count and were more likely to have a history of other AIDS‐defining illnesses at cohort entry. Twenty‐five percent and 10% of PWH with and without KS, respectively, died during follow‐up. When analyses were restricted to only the 20 229 ART‐naïve patients, the overall baseline characteristics were similar: 38% were from Brazil, the median age was 34 years, 45% were MSM. and the median CD4 cell count was 230 cells/μL. A total of 200 ART‐naïve PWH were diagnosed with KS and 9% died.

**Table 1:** Descriptive characteristics of all patients with and without KS diagnosis after clinic entry

	KS (N = 481)	No KS (N = 25 500)	Total (N = 25 981)	*p* value^a^
Site, n (%)				
Argentina	12 (2)	1423 (6)	1435 (6)	<0.001
CCASAnet Brazil	177 (37)	5298 (21)	5475 (21)	
Chile	129 (27)	3575 (14)	3704 (14)	
Coorte Brasil	23 (5)	5103 (20)	5126 (20)	
Honduras	8 (2)	1367 (5)	1375 (5)	
Mexico	51 (11)	1947 (8)	1998 (8)	
Peru	81 (18)	6787 (25)	6868 (26)	
Age at clinic entry	34 [29 to 41]	34 [28 to 42]	34 [28 to 42]	0.73
Sex/sexual risk factor for HIV transmission^b^				
Heterosexual men	70 (15)	5621 (22)	5691 (22)	
Heterosexual women	17 (4)	5523 (22)	5540 (21)	
Men who have sex with men	344 (72)	10 924 (43)	11 268 (43)	
Other/unknown men	40 (8)	2395 (9)	2435 (9)	
Other/unknown women	10 (2)	1035 (4)	1045 (4)	
History of injection drug use	3 (1)	302 (1)	305 (1)	0.26
Year of clinic entry, median [IQR]	2010 [2005 to 2013]	2010 [2006 to 2014]	2010 [2006 to 2014]	0.045
CD4 cell count (cells/μL)^c^	80 [29 to 195]	240 [95 to 405]	237 [92 to 402]	<0.001
History of AIDS‐defining illness^d^	109 (23)	3532 (14)	3641 (14)	<0.001
Died during follow‐up	122 (25)	2595 (10)	2717 (10)	<0.001

ART, antiretroviral therapy; IQR, interquartile range; KS, Kaposi’s sarcoma.

^a^ Tests used: Pearson test for categorical variables and Wilcoxon test for continuous variables

^b^ other/unknown sexual risk factors included individuals with unknown HIV transmission factors and those with injection drug use, perinatal infection, haemophilia, or other potential exposures identified

^c^ total of 21 823 persons with CD4 cell count available within 6 months before or after date of clinic entry (16% missing)

^d^ history of AIDS‐defining illness other than KS diagnoses before or at clinic entry.

### 3.2 Incidence trends over time

**Figure 1:**
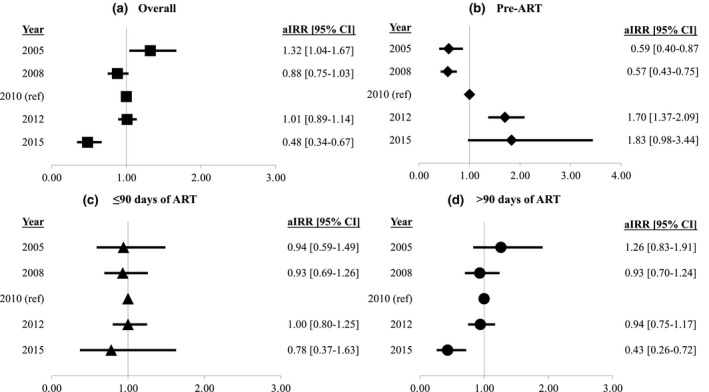
Adjusted incidence rate ratios of calendar year (reference: 2010). In addition to calendar year, multivariable models included clinical site, age at cohort entry, sex/sexual route of HIV infection, CD4 cell count at cohort entry and prevalent AIDS‐defining illness (excluding KS) at cohort entry. (**A**) Incidence trend including all person‐time. (**B**) Incidence trend restricting to person‐time before ART initiation. (**C**) Incidence trend restricting person‐time to the first 90 days after ART initiation. (**D**) Incidence trend restricting person‐time to more than 90 days following ART initiation

The incidence of KS decreased from 55.1 in 2000 to 3.0 per 1000 person‐years in 2017. This decrease largely occurred from 2000 to 2005. Incidence remained relatively stable between 3 and 5 per 1000 person‐years from 2005 to 2017 (Figure S1). We examined incidence over time that accounted for temporal changes in patient demographics (age, sex/sexual risk factor for HIV transmission, cohort site) and clinical characteristics at clinic entry (CD4 cell count and history of other AIDS‐defining illness) using multivariable models (Figure 1). As shown in Figure 1A, the relative risk of KS was higher in 2005 compared to 2010 after adjusting for the above confounders but was similar in 2008, 2012 and 2015 (adjusted incidence rate ratios all close to 1.0). We analysed incidence trends in KS among ART‐naïve PWH according to person‐time relative to ART initiation. Figure 1B demonstrates lower KS risk in 2005 and 2008 compared to 2010 during the period before ART initiation but higher risk during the period before ART initiation in later years. The risk of KS was also higher in 2015 relative to 2010 in the first 90 days after ART initiation (Figure 1C). Conversely, the risk of KS more than 90 days following ART decreased during the study period (Figure 1D).

### 3.3 Characteristics associated with risk of KS

We examined characteristics associated with KS risk using Cox proportional hazard models among patients who were ART‐naïve upon clinic entry (n = 20 229). Among the 371 KS diagnoses, 200 occurred before ART initiation and 171 occurred after ART initiation. Table 2 describes the results of the multivariable models for KS risk before and following ART initiation. During both periods, sex/sexual risk factor for HIV transmission was highly associated with KS, with heterosexual men and women having significantly decreased hazards compared to MSM. KS risk was strongly associated with low CD4 cell count both before and after ART initiation. Following ART initiation, age and prior history of other AIDS‐defining illnesses at ART initiation were also associated with increased risk of KS. Adjusted log hazard ratios for risk of KS before and after ART by CD4 cell count and age are shown in Figures S2 and S3 respectively.

**Table 2:** Multivariable Cox proportional hazard models^a^ for patient characteristics associated with KS risk before and after ART initiation^b^

	Hazard for KS before ART initiation		Hazard for KS after ART initiation	
	aHR [95% CI]	*p* value	aHR [95% CI]	*p* value
Age at clinic entry or ART initiation^c^, ^d^				
20 years	0.76 [0.46 to 1.25]	0.484	0.53 [0.29 to 0.96]	0.016
25 years	0.85 [0.63 to 1.15]		0.70 [0.49 to 1.00]	
30 years	0.95 [0.84 to 1.06]		0.88 [0.76 to 1.02]	
35 years (reference)	1		1	
40 years	0.99 [0.92 to 1.07]		0.94 [0.86 to 1.04]	
45 years	0.94 [0.78 to 1.13]		0.77 [0.61 to 0.96]	
50 years	0.87 [0.63 to 1.21]		0.59 [0.39 to 0.89]	
Sex/sexual risk factor for HIV transmission				
Men who have sex with men (reference)	1	<0.001	1	<0.001
Heterosexual men	0.31 [0.20 to 0.47]		0.48 [0.32 to 0.72]	
Heterosexual women	0.12 [0.06 to 0.25]		0.09 [0.04 to 0.23]	
Other/unknown men	0.47 [0.25 to 0.87]		0.65 [0.33 to 1.30]	
Other/unknown women	0.27 [0.08 to 0.87]		0.40 [0.12 to 1.30]	
CD4 cell count at clinic entry or ART initiation^c^, ^e^				
50 cells/μL	2.53 [1.99 to 3.21]	<0.001	1.90 [1.47 to 2.45]	<0.001
100 cells/μL	1.74 [1.55 to 1.96]		1.53 [1.33 to 1.76]	
200 cells/μL (reference)	1		1	
350 cells/μL	0.56 [0.47 to 0.68]		0.52 [0.37 to 0.73]	
500 cells/μL	0.36 [0.23 to 0.56]		0.29 [0.13 to 0.66]	
HIV RNA at clinic entry or ART initiation	1.16 [0.95 to 1.41]	0.150	0.95 [0.79 to 0.66]	0.589
(per 1 unit increase in log_10_ RNA)				
History of AIDS‐defining illness at clinic entry or ART initiation	1.18 [0.80 to 1.74]	0.405	1.72 [1.23 to 2.40]	0.002
Initiation of PI‐ containing ART regimen			1.16 [0.78 to 1.73]	0.462

aHR, adjusted hazard ratio; ART, antiretroviral therapy; CI, confidence interval; KS, Kaposi’s sarcoma; PI, protease inhibitor.

^a^ Proportional hazard models restricted to patients ART‐naïve at clinic entry and were stratified by country to account for geographic differences

^b^ models run from time clinic and from ART initiation to KS, death, censoring. Age, CD4 cell count, log_10_ HIV RNA and history of other AIDS‐defining illness were all relative to either date of clinic entry or date of ART for the two separate models respectively

^c^ age and CD4 fit with restricted cubic splines. Results presented with selected values in comparison to reference value

^d^ tests for non‐linearity *p*‐value for age: 0.23 and 0.005 respectively

^e^ tests for non‐linearity *p*‐value for CD4: <0.001 and 0.17 respectively.

### 3.4 Mortality risk after KS

**Figure 2:**
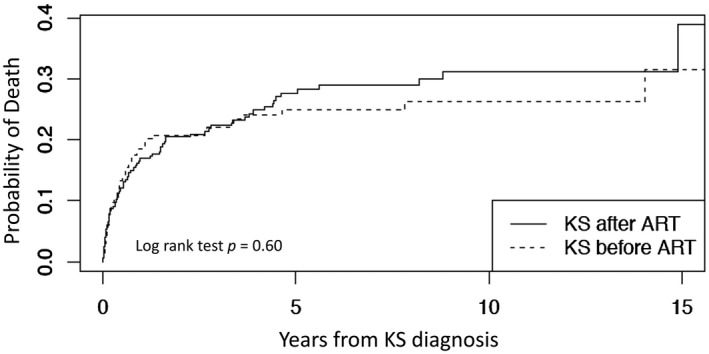
Kaplan–Meier curve of mortality risk after KS by timing of diagnosis relative to ART initiation

Lastly, we evaluated mortality risk following KS diagnosis relative to occur before or after ART initiation. We included all 481 KS diagnoses of which 200 (42%) occurred before ART initiation and 281 (58%) occurred at any point after ART initiation. Patient characteristics at the time of KS diagnosis are shown in Table S1. The probability of death was similarly high between those diagnosed with KS before or after ART initiation; approximately 25% of persons diagnosed with KS died within five years (Figure 2). In adjusted models (Table 3) the hazard for mortality was 23% lower among those PWH with KS diagnosed before ART initiation but this result was not statistically significant. Only older age and low CD4 cell count at KS diagnosis remained strongly associated with risk of death in adjusted models. The calendar year of KS diagnosis did not predict mortality risk. Adjusted log hazard ratios for risk of mortality following KS by CD4 cell count and age are shown in Figure S4.

**Table 3:** Univariate and multivariable Cox proportional hazard models^a^ for patient characteristics associated risk of death following KS diagnosis

	Unadjusted hazard ratio [95% CI]	*p* value	Adjusted hazard ratio [95% CI]	*p* value
KS diagnosis before ART initiation	0.88 [0.61 to 1.27]	0.488	0.77 [0.51 to 1.18]	0.231
Age at KS diagnosis^b^, ^c^				
20 years	0.59 [0.44 to 0.80]	0.001	0.86 [0.38 to 1.96]	0.001
25 years	0.71 [0.58 to 0.86]		0.89 [0.53 to 1.48]	
30 years	0.84		0.92 [0.74 to 1.13]	
35 years (reference)	1		1	
40 years	1.19 [1.08 to 1.31]		1.20 [1.08 to 1.32]	
45 years	1.42 [1.16 to 1.73]		1.52 [1.21 to 1.91]	
50 years	1.69 [1.25 to 2.27]		1.97 [1.33 to 2.93]	
Sex/sexual risk factor for HIV transmission				
Men who have sex with men (reference)	1	0.118	1	0.204
Heterosexual men	1.11 [0.66 to 1.87]		0.99 [0.58 to 1.70]	
Heterosexual women	2.67 [1.29 to 5.51]		2.29 [1.10 to 4.79]	
Other/unknown men	1.41 [0.68 to 2.91]		1.36 [0.63 to 2.96]	
Other/unknown women	1.11 [0.27 to 4.57]		0.61 [0.15 to 2.55]	
CD4 cell count at KS diagnosis^b^, ^d^				
50 cells/μL	1.36 [1.19 to 1.54]	<0.001	2.02 [1.53 to 2.66]	<0.001
100 cells/μL (reference)	1		1	
200 cells/μL	0.65 [0.54 to 0.78]		1.33 [1.10 to 1.61]	
350 cells/μL	0.41 [0.28 to 0.60]		0.92 [0.66 to 1.28]	
500 cells/μL	0.28 [0.16 to 0.48]		0.88 [0.47 to 1.65]	
HIV RNA at KS diagnosis (per 1 unit increase in Log_10_ RNA)	1.08 [0.90 to 1.28]	0.424	1.03 [0.84 to 1.27]	0.757
Year of KS diagnosis				
2006	1.03 [0.91 to 1.17]	0.656	0.98 [0.86 to 1.12]	0.813
2009 (reference)	1		1	
2012	0.97 [0.85 to 1.10]		1.02 [0.89 to 1.16]	

ART, antiretroviral therapy; CI, confidence interval; KS, Kaposi’s sarcoma.

^a^ Models stratified by country to account for geographic/site differences

^b^ age and CD4 fit with restricted cubic splines. Results presented with selected values in comparison to reference value

^c^ test for non‐linearity *p*‐value for age: 0.002

^d^ test for non‐linearity *p*‐value for CD4: <0.001.

## 4 DISCUSSION

In this large, multinational cohort of PWH in Latin America, we observed both stable and dynamic trends in KS epidemiology, risk factors and outcomes in the current ART era. While the incidence of KS has decreased in the region since 2000, most of this occurred in the early years and the overall risk of KS has remained fairly stable since 2005 to 2008. After accounting for other factors, the risk of KS before and shortly after ART initiation appears to have increased in recent years, whereas the incidence >90 days after ART initiation has decreased, perhaps a result of improved long‐term adherence and HIV outcomes. Immunodeficiency and MSM HIV transmission risk factor were consistently associated with KS risk in the region. Lastly, the risk of mortality following KS (approximately 25% within five years after KS diagnosis) was statistically similar for those diagnosed before or after ART initiation and was unchanged over time, showing that ongoing work is needed to improve long‐term outcomes of PWH with KS in the region.

The incidence of KS dramatically decreased in PWH in Latin America in the early 2000s. In many high‐income countries, rates of KS fell significantly in the early 1990s, though KS remains an important cause of cancer morbidity in the US and other countries [13, 14, 15]. A recent global study of KS showed that among PWH initiated on ART and accounting for sex and CD4 cell count, the incidence of KS was similar in Latin America compared to Europe and North America [4]. However, this study included observation time before ART initiation and showed a relative increase in KS incidence over time in the period before ART initiation in the region, even adjusting for immunodeficiency and HIV transmission risk factors. In low‐ and middle‐income countries, KS remains one of the most frequent cancers diagnosed in PWH despite global expansion of ART access [16, 17, 18, 19]. While regional differences in immunodeficiency and HHV‐8 prevalence contribute to differences, reasons for differences in KS epidemiology among PWH are not fully understood [4, 20]. In this study, KS incidence decreased in the early 2000s overall and after accounting for concurrent changes in CD4 cell count, country and other patient characteristics at enrolment. This drop in KS incidence was reflective of the introduction of ART to PWH with CD4 cell count <200 cells/μL which occurred in 1996 in Brazil, Mexico and Argentina and 2000 to 2004 in the other countries included in our study. From 2004 until 2014, treatment guidelines in the region incrementally increased the CD4 cell threshold until the treat‐all era began in 2014 to 2015 for countries included in this study. Notably, the KS rate and risk remained stable from 2010 to 2017, demonstrating an ongoing burden of disease that could be mitigated by early HIV diagnosis and engagement in care. Our analysis also demonstrated changes in relative risk in relation to ART timing: increasing risk over time in the period before and shortly after ART initiation and decreasing risk ≥90 days after ART initiation. These changes may be in part an artifact due to decreasing pre‐ART and increasing post‐ART person‐time because of earlier ART initiation given changes in treatment guidelines based upon CD4 cell count or due to improvement in long‐term virological control following ART initiation over time with improved tolerability of newer medications. The relative increase in KS diagnoses in the period shortly after ART initiation may be a reflection of earlier ART initiation and diagnosis of KS shortly after initiation. It also could reflect an increase in KS diagnoses as a result of unmasking by ART due to immune reconstitution inflammatory syndrome (IRIS), which has been reported with the use of newer ART including dolutegravir [21, 22].

We observed differences in patient risk factors for KS relative to ART initiation. Before and after ART initiation, sex/sexual risk factor for HIV transmission and immune deficiency were strongly associated with KS. After ART initiation, age and history of other AIDS‐defining illnesses were also associated with risk. Numerous studies have shown higher rates of KS among MSM and those with low CD4 count [23, 24]. However, a global study from IeDEA highlighted that among PWH started on ART, the associations between sex and CD4 cell count on KS risk is not uniform among all regions [4]. We observed a decreased risk of KS following ART initiation among younger and older PWH. Studies of MSM with HIV in the United States also showed a decreased risk of KS with older age but noted increased risk among older men who did not use ART [25]. Our results may reflect the lower risk of virologic failure and higher rates of ART adherence observed among older adults or differences in the underlying HHV‐8 epidemiology [26].

We observed a high risk of death following KS diagnosis, but no difference between PWH diagnosed with KS before versus after ART initiation. Despite improved survival among PWH, the history of KS has been associated with long‐term risk of mortality [7, 9]. In general, KS treatment is associated with better outcomes among ART‐naïve patients, than among ART‐experienced patients [27]. Other factors that may contribute to KS risk after ART initiation (such as treatment interruption or lack of virological suppression) may also contribute to poor KS outcomes in these patients. Outcomes among patients diagnosed with KS after ART initiation also could differ if KS is due to IRIS, which is generally associated with a worse prognosis [28]. In adjusted models, only older age and low CD4 cell count at KS diagnosis were significantly associated with mortality risk, underscoring the importance of early HIV diagnosis and treatment to improve not only rates of AIDS‐defining conditions such as KS but also their outcomes. We also observed a higher risk of mortality in heterosexual women compared to MSM (albeit with a wide confidence interval given the small number of women with KS). Previous analyses of our cohort have shown higher mortality in women compared to MSM [29]. However, our observed sex disparity in mortality after KS specifically warrants additional investigation. Lastly, despite advancements in HIV and KS treatments over time, our analyses showed no difference in risk of mortality by year of KS diagnosis, underscoring the ongoing importance of KS as a risk factor for death in PWH.

This study has important limitations to consider. Our study was strengthened by careful validation of KS diagnoses. However, the lack of information regarding KS stage (visceral vs. mucocutaneous) and accompanying symptoms or progression (to evaluate for IRIS) precluded evaluating disease‐specific characteristics and outcomes. Furthermore, without corresponding KS staging, our KS treatment data could not be reliably used in analyses. Many CCASAnet sites are tertiary referral clinics and the patients may not be representative of all PWH in their respective cities and countries. Two sites in Brazil (Instituto Nacional de Infectologia and AIDS Reference and Training Center) are referral centres for KS, specifically. We excluded patients with KS diagnoses prior to clinic entry and adjusted for site to reduce this bias. Furthermore, our results are only reflective of KS epidemiology of PWH without diagnosed KS at the time of clinic entry and exclude patients whose KS prompted HIV diagnosis and entry into care. This also meant we excluded patients with a second episode of KS after entry into care, who may have different outcomes. Our study was limited by lack of HHV‐8 seroprevalence data, and thus, we cannot describe how HHV‐8 epidemiology contribute to our results. Of note, studies from Brazil and Peru suggest that HHV‐8 prevalence is higher among PWH and MSM [30, 31]. Our data are also limited by a lack of cause of death information to inform long‐term outcomes of PWH and KS. Lastly, HIV treatment recommendations changed during the study period with only recent adoption of treat‐all policies in all study sites. It is possible that some of the positive effects of the treat‐all era on KS trends will be more noticeable in the years after our study period.

## 5 CONCLUSIONS

In conclusion, we observed dynamic changes in KS epidemiology among clinical cohorts from Latin America. With the increasing availability of ART, the incidence of KS has dramatically decreased since 2000, even after controlling for CD4 cell count at clinic entry and other patient factors. However, we observed stable rates of KS from approximately 2008 to 2017, highlighting the ongoing need to reduce KS. Currently, our best tools for reducing KS are increasing early HIV diagnosis and treatment. The risk of KS remains highest before and shortly after ART initiation and is highest among MSM and patients with severe immunodeficiency. Mortality following KS diagnosis was not associated with the year of diagnosis in adjusted models. Further research in KS treatment interventions, long‐term morbidity, and specific causes of death of PWH with KS are needed to improve patient outcomes.

## COMPETING INTEREST

All authors of this study declare they have no competing interests.

## AUTHORS’ CONTRIBUTIONS

All authors have read and approved the final manuscript. J.L.C., C.C.M., B.E.S., B.G., B.R.C., P.F.B.Z. and V.F. designed the research study. B.G., E.G., V.F., D.P., P.F.B.Z., B.C.R., M.M.E., R.A.S., S.B.T., S.R.P., M.L.R.I, P.R.A., U.T., C.B., E.L., J.N., C.P.C. and A.G. contributed essential research contributions to the collection and validation of high‐quality observational data. J.L.C., C.C.M., B.E.S., A.K., C.A.J., B.R.C., P.F.B.Z. and V.F. wrote the manuscript.

## Supporting information


**Figure S1.** Incidence of KS by year, 2000 to 2017.

**Figure S2.** Adjusted log hazard ratio for KS before ART by (a) CD4 cell count and (b) age. Model (a) adjusted relative to an individual aged 35 years from Brazil, MSM, median log_10_ HIV RNA and no other AIDS‐defining illness before enrolment. Model (b) adjusted relative to an individual from Brazil, MSM, CD4 cell count of 200 cells/μL, median log_10_ HIV RNA and no other AIDS‐defining illness before enrolment.

**Figure S3.** Adjusted log hazard ratio for KS after ART by (a) CD4 cell count and (b) age. Model (a) adjusted relative to an individual aged 35 years from Brazil, MSM, median log_10_ HIV RNA and no other AIDS‐defining illness before enrolment. Model (b) adjusted relative to an individual from Brazil, MSM, CD4 cell count of 200 cells/μL, median log_10_ HIV RNA and no other AIDS‐defining illness before enrolment.

**Figure S4.** Adjusted log hazard ratio for mortality after KS by (a) CD4 cell count and (b) age. Model (a) adjusted relative to an individual aged 35 years from Brazil, MSM, median log_10_ HIV RNA, KS diagnosis in 2011 and KS after ART initiation. Model (b) adjusted relative to an individual from Brazil, MSM, CD4 cell count of 100 cells/μL, median log_10_ HIV RNA, KS diagnosis in 2011 and KS after ART initiation.

**Table S1.** Descriptive characteristics at all patients with KS diagnosis following clinic entry
