# Supplementary figures and images for: Antiretroviral therapy and Kaposi’s sarcoma trends and outcomes among adults with HIV in Latin America
Source: J Int AIDS Soc. 2021 Jan 6;24(1):e25658. doi: 10.1002/jia2.25658 (PMC7787071; doi:10.1002/jia2.25658)

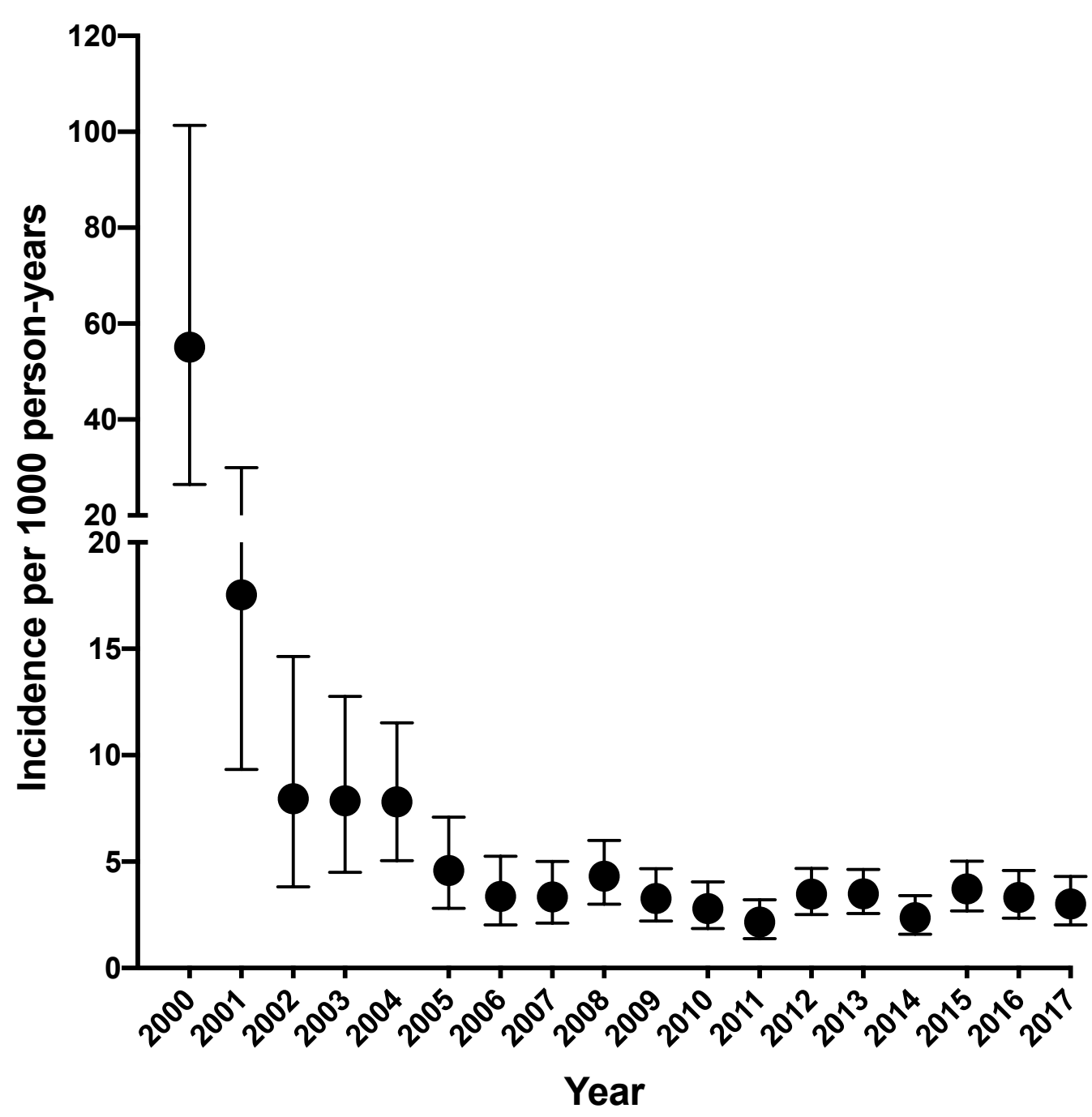

Supplement: Supplementary file 1 — Figure S1. Incidence of KS by year, 2000 to 2017. [file JIA2-24-e25658-s001.pdf]

a.

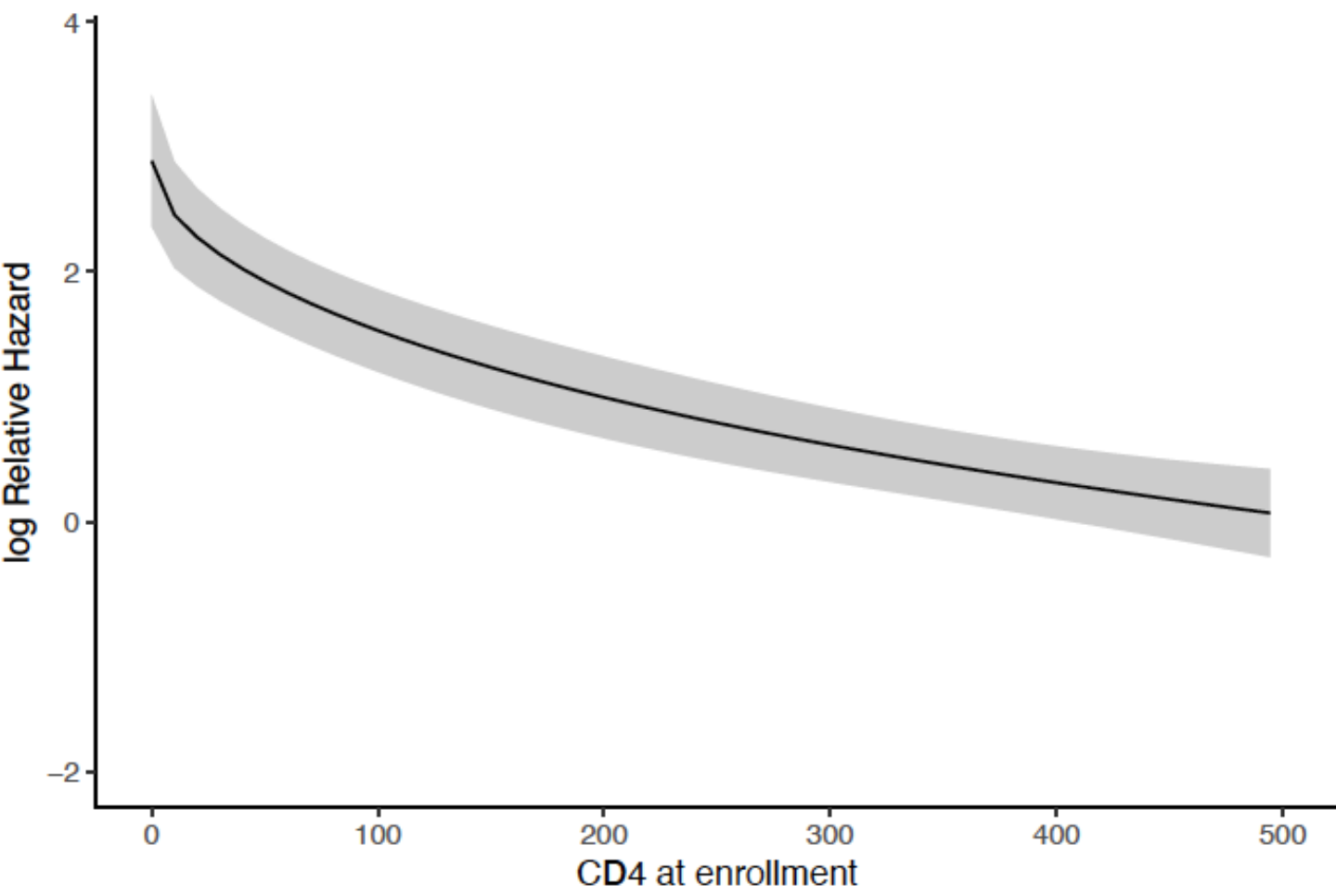

b.

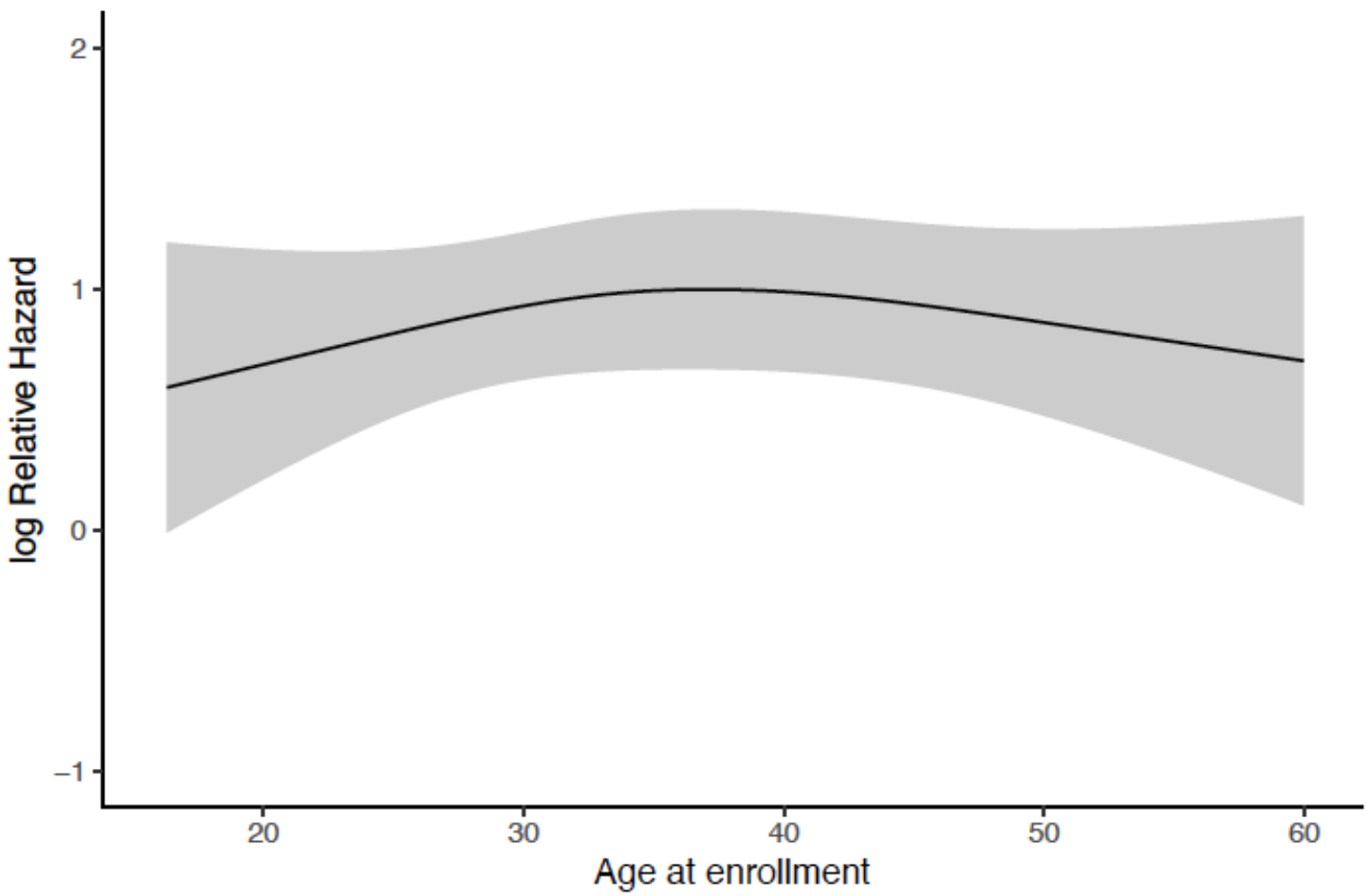

Supplement: Supplementary file 2 — Figure S2. Adjusted log hazard ratio for KS before ART by (a) CD4 cell count and (b) age. Model (a) adjusted relative to an individual aged 35 years from Brazil, MSM, median log10 HIV RNA and no other AIDS‐defining illness before enrolment. Model (b) adjusted relative to an individual from Brazil, MSM, CD4 cell count of 200 cells/μL, median log10 HIV RNA and no other AIDS‐defining illness before enrolment. [file JIA2-24-e25658-s002.pdf]

a.

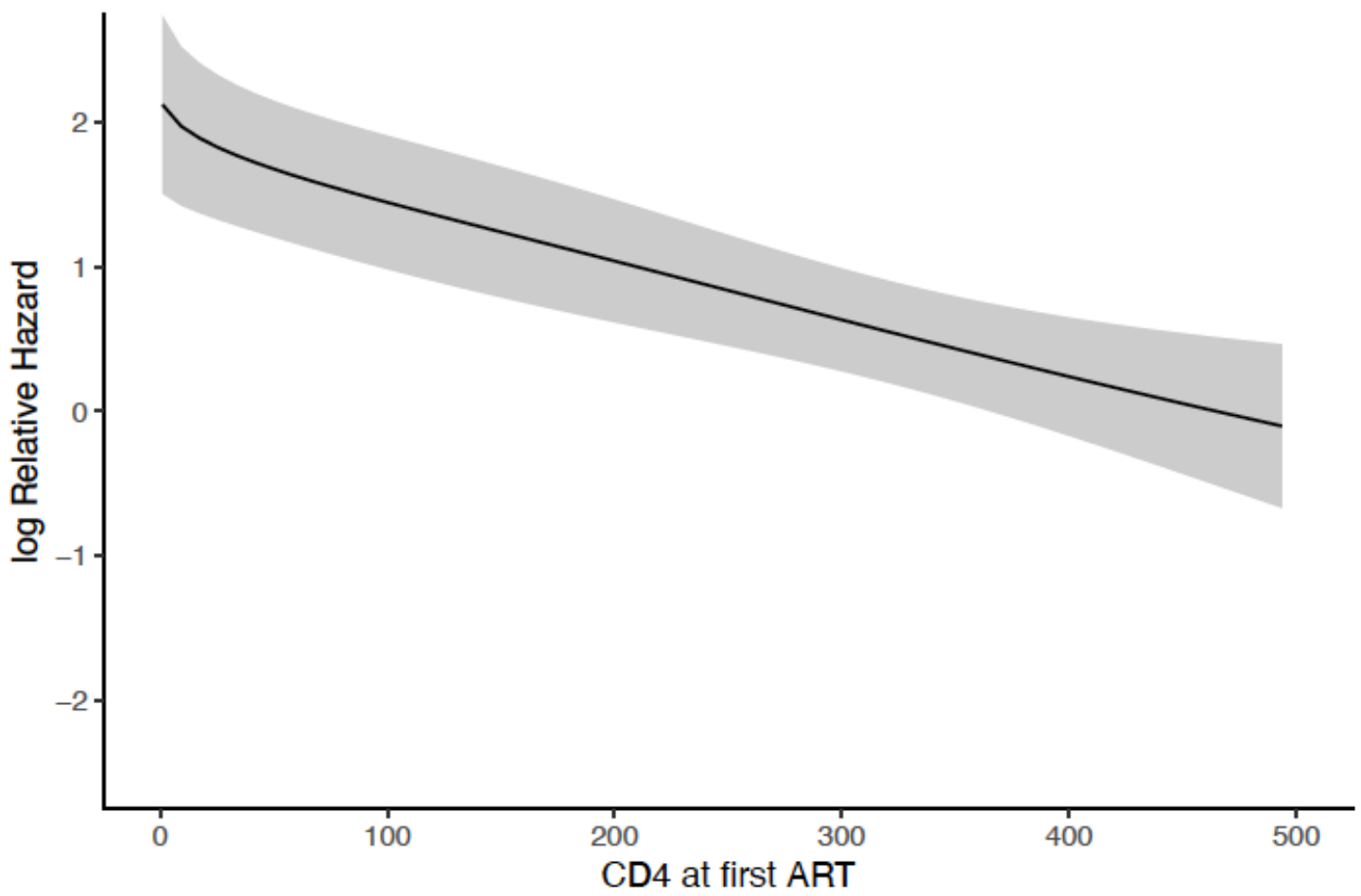

b.

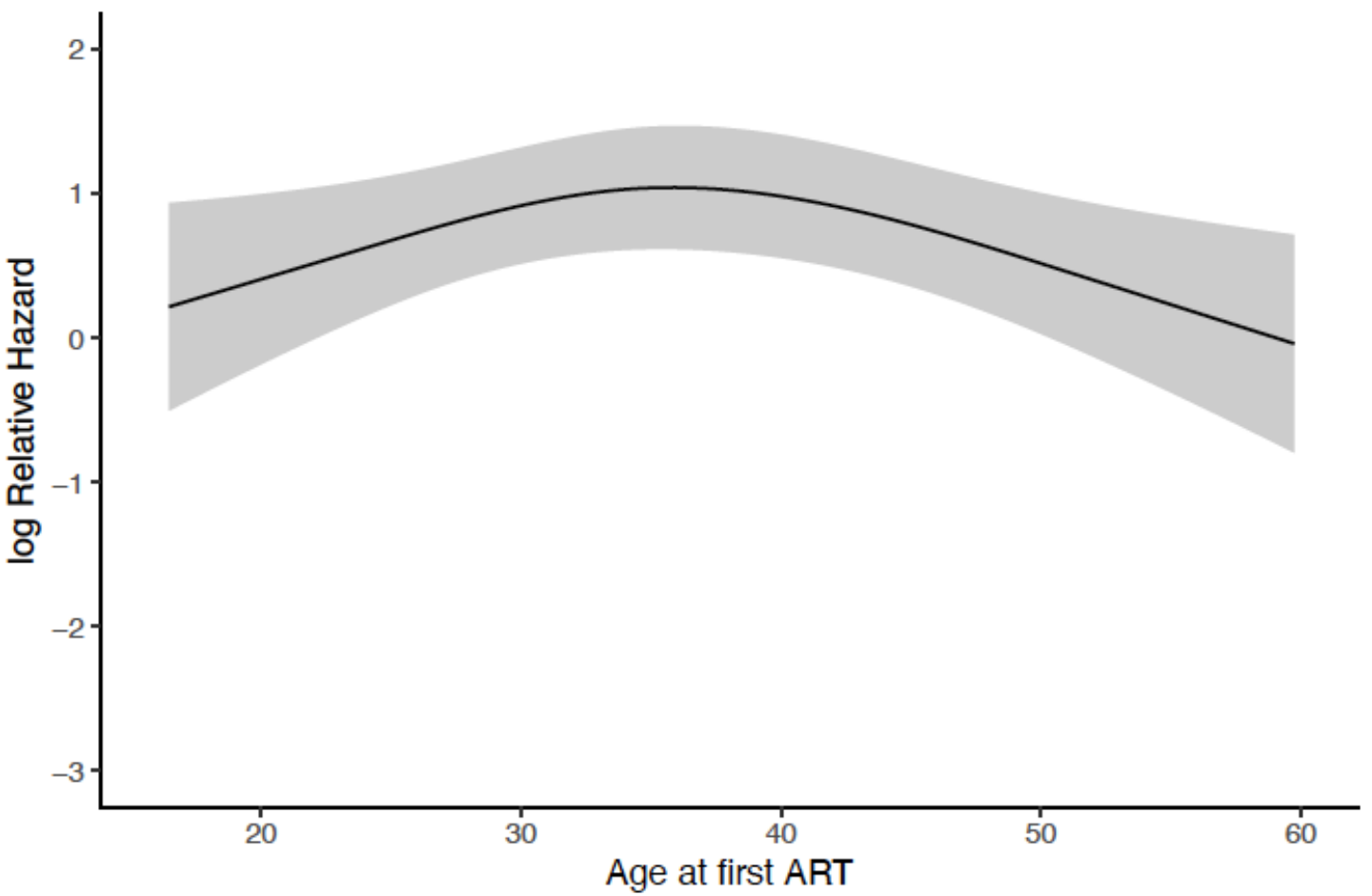

Supplement: Supplementary file 3 — Figure S3. Adjusted log hazard ratio for KS after ART by (a) CD4 cell count and (b) age. Model (a) adjusted relative to an individual aged 35 years from Brazil, MSM, median log10 HIV RNA and no other AIDS‐defining illness before enrolment. Model (b) adjusted relative to an individual from Brazil, MSM, CD4 cell count of 200 cells/μL, median log10 HIV RNA and no other AIDS‐defining illness before enrolment. [file JIA2-24-e25658-s003.pdf]

a.

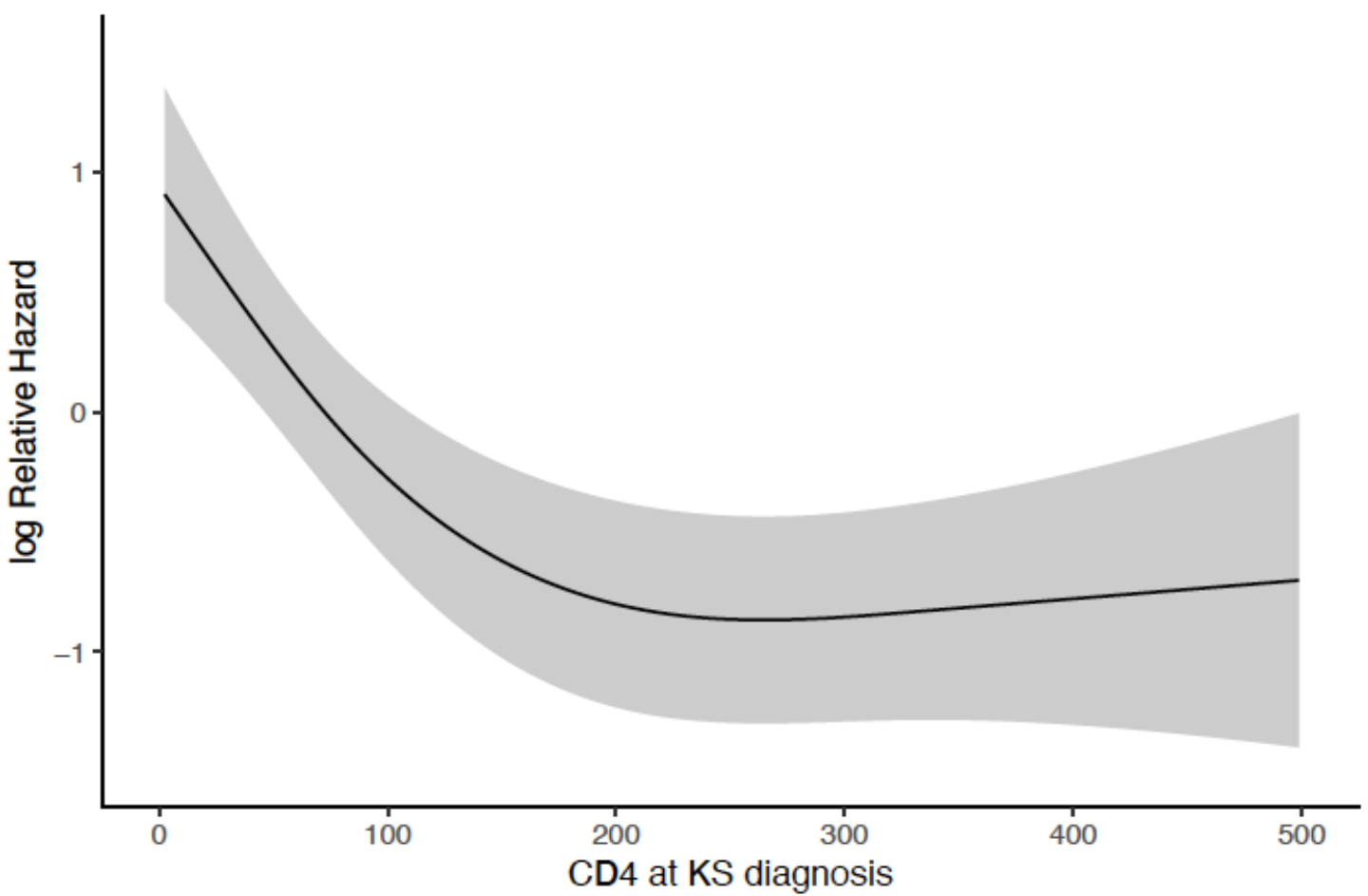

b.

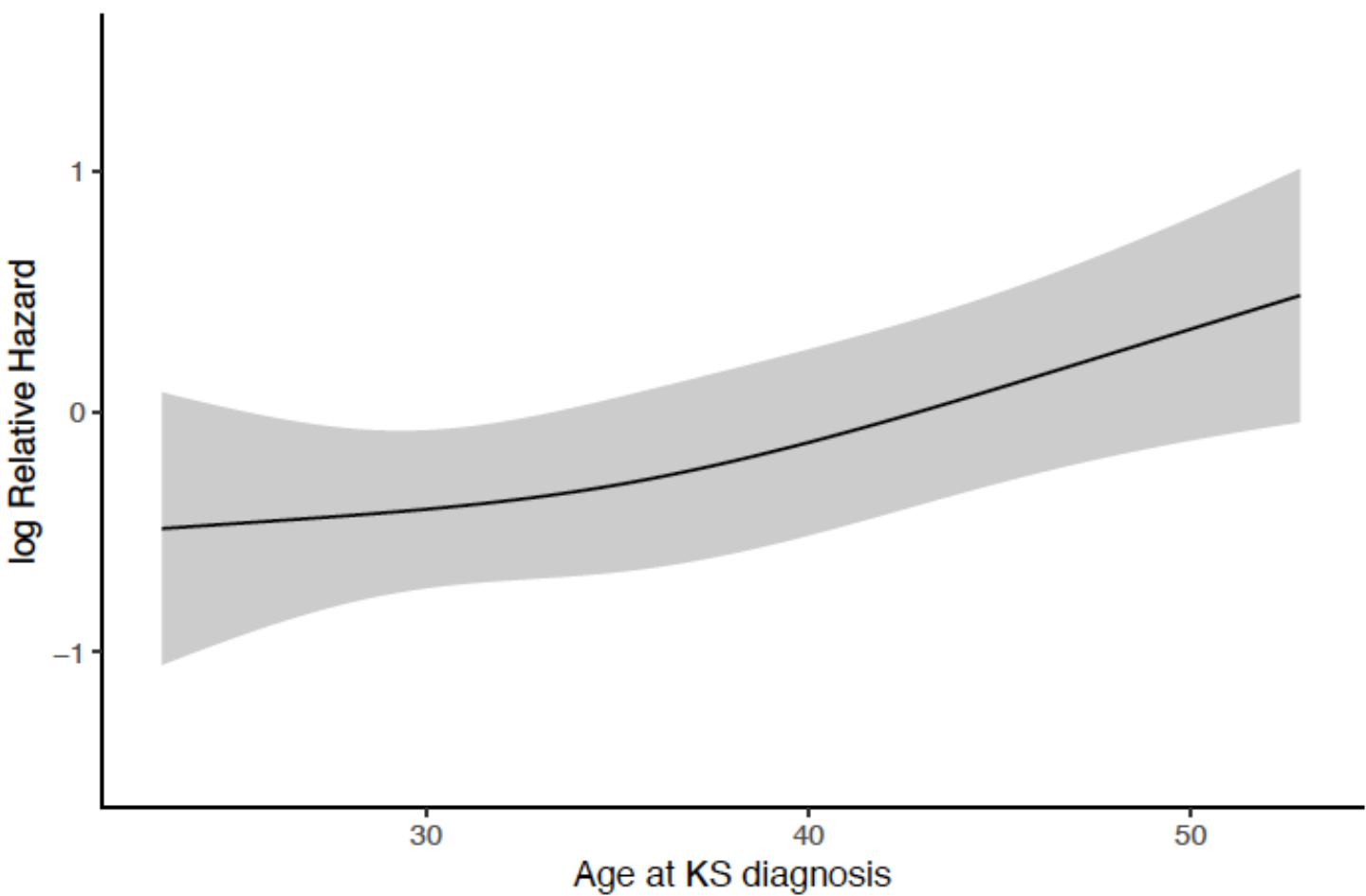

Supplement: Supplementary file 4 — Figure S4. Adjusted log hazard ratio for mortality after KS by (a) CD4 cell count and (b) age. Model (a) adjusted relative to an individual aged 35 years from Brazil, MSM, median log10 HIV RNA, KS diagnosis in 2011 and KS after ART initiation. Model (b) adjusted relative to an individual from Brazil, MSM, CD4 cell count of 100 cells/μL, median log10 HIV RNA, KS diagnosis in 2011 and KS after ART initiation. [file JIA2-24-e25658-s004.pdf]
