# Supplementary material for: Antiretroviral therapy and Kaposi’s sarcoma trends and outcomes among adults with HIV in Latin America
Source: J Int AIDS Soc. 2021 Jan 6;24(1):e25658. doi: 10.1002/jia2.25658 (PMC7787071; doi:10.1002/jia2.25658)
Supplement: Supplementary file 5 — Table S1. Descriptive characteristics at all patients with KS diagnosis following clinic entry [file JIA2-24-e25658-s005.docx]

Supplemental Table 1. Descriptive characteristics at all patients with KS diagnosis following clinic entry

|  | KS before ART  N=200 | KS after ART  N=281 | Total  N=481 | *P* value^a^ |
| --- | --- | --- | --- | --- |
| Site, n (%)  Argentina  Brazil  Chile  Honduras  Mexico  Peru | 4 (2)  76 (38)  63 (32)  4 (2)  14 (7)  39 (20) | 8 (3)  124 (44)  66 (23)  4 (1)  37 (13)  42 (15) | 12 (2)  200 (42)  129 (27)  8 (2)  51 (11)  81 (17) | 0.074 |
| Age at KS diagnosis in years, median [IQR] | 33 [28-41] | 35 [29-41] | 34 [29-41] | 0.330 |
| Sex / sexual risk factor for HIV transmission  Heterosexual men  Heterosexual women  Men who have sex with men  Other/unknown men  Other/unknown women | 28 (14)  8 (4)  149 (74)  12 (6)  3 (1) | 42 (15)  9 (3)  195 (69)  28 (10)  7 (2) | 70 (15)  17 (4)  344 (72)  40 (8)  10 (2) | 0.483 |
| History of injection drug use | 0 (0) | 3 (1) | 3 (1) | 0.143 |
| Year of KS diagnosis | 2011 [2007-2014] | 2011 [2007-2014] | 2011 [2007-2014] | 0.986 |
| CD4 cell count at KS diagnosis (cells/μL)^b^ | 76 [28-173] | 86 [34-190] | 85 [33-186] | 0.261 |
| Log_10_ HIV RNA at KS diagnosis^c^ | 5.15 [4.68-5.67] | 3.61 [2.60-5.00] | 4.71 [2.60-5.34] | <0.001 |
| Follow-up time in years until death or censoring | 3.80 [1.10-8.53] | 3.91 [1.33-7.81] | 3.89 [1.24-8.09] | 0.626 |
| Lost to follow-up | 25 (12) | 48 (17) | 73 (15) | 0.167 |
| Died during follow-up | 48 (24) | 74 (26) | 122 (25) | 0.562 |
| KS treatment^d^  ART  Chemotherapy  ART + chemotherapy  ART + chemotherapy + other  ART + other  Other  Unknown | 89 (45)  11 (6)  66 (33)  0 (0)  4 (2)  1 (1)  29 (14) | 191 (68)  0 (0)  86 (31)  2 (1)  2 (1)  0 (0)  0 (0) | 280 (58)  11 (2)  152 (32)  2 (1)  6 (1)  1 (0)  29 (6) | <0.001 |

^a^ Tests used: Pearson test for categorical variables and Wilcoxon test for continuous variables

^b^ Total of 405 persons with CD4 cell count available within 6 months before to 30 days after date of KS diagnosis (16% missing)

^c^ Total of 372 persons with HIV RNA available within 6 months before to 30 days after date of KS diagnosis (23% missing)

^d^ Total of 452 persons with KS treatment record available (6% missing). Other KS treatments included radiation therapy and surgery.

Abbreviations used:

KS: Kaposi’s sarcoma

IQR: interquartile range

ART: antiretroviral therapy
